# Technology-Supported Self-Guided Nutrition and Physical Activity Interventions for Adults With Cancer: Systematic Review

**DOI:** 10.2196/12281

**Published:** 2019-02-12

**Authors:** Nicole Kiss, Brenton James Baguley, Kylie Ball, Robin M Daly, Steve F Fraser, Catherine L Granger, Anna Ugalde

**Affiliations:** 1 Institute for Physical Activity and Nutrition Deakin University Geelong Australia; 2 Department of Cancer Experiences Research Peter MacCallum Cancer Centre Melbourne Australia; 3 School of Psychology Faculty of Health Deakin University Burwood Australia; 4 School of Human Movement and Nutrition Sciences The University of Queensland St Lucia Australia; 5 School of Physiotherapy Faculty of Medicine, Dentistry and Health Sciences University of Melbourne Melbourne Australia; 6 Department of Physiotherapy The Royal Melbourne Hospital Melbourne Australia; 7 School of Nursing and Midwifery Faculty of Health Deakin University Burwood Australia

**Keywords:** cancer, diet, exercise, nutrition, physical activity, self-guided interventions, technology

## Abstract

**Background:**

Nutrition and physical activity interventions are important components of cancer care. With an increasing demand for services, there is a need to consider flexible, easily accessible, and tailored models of care while maintaining optimal outcomes.

**Objective:**

This systematic review describes and appraises the efficacy of technology-supported self-guided nutrition and physical activity interventions for people with cancer.

**Methods:**

A systematic search of multiple databases from 1973 to July 2018 was conducted for randomized and nonrandomized trials investigating technology-supported self-guided nutrition and physical activity interventions. Risk of bias was assessed using the Cochrane Risk of Bias tool. Outcomes included behavioural, health-related, clinical, health service, or financial measures.

**Results:**

Sixteen randomized controlled trials representing 2684 participants were included. Most studies were web-based interventions (n=9) and had a 12-week follow-up duration (n=8). Seven studies assessed dietary behaviour, of which two reported a significant benefit on diet quality or fruit and vegetable intake. Fifteen studies measured physical activity behaviour, of which eight studies reported a significant improvement in muscle strength and moderate-to-vigorous physical activity. Four of the nine studies assessing the health-related quality of life (HRQoL) reported a significant improvement in global HRQoL or a domain subscale. A significant improvement in fatigue was found in four of six studies. Interpretation of findings was influenced by inadequate reporting of intervention description and compliance.

**Conclusions:**

This review identified short-term benefits of technology-supported self-guided interventions on the physical activity level and fatigue and some benefit on dietary behaviour and HRQoL in people with cancer. However, current literature demonstrates a lack of evidence for long-term benefit.

**Trial Registration:**

PROSPERO CRD42017080346; https://www.crd.york.ac.uk/prospero/display_record.php?RecordID=80346

## Introduction

It is estimated that over 32 million people are living with cancer worldwide [1], and the predicted global incidence of cancer is estimated to increase from 14 million new cases in 2012 to more than 17 million in 2020 [1]. Cancer is now considered a chronic disease, and the number of cancer survivors in the United States is estimated to exceed 20 million by 2026 [2]. This rapid increase is adding pressure on health care systems to cope with the growing number of people requiring treatment while maintaining high-quality health care. As a result, there is a need to consider alternative, easily accessible, and flexible models of delivering care, in particular, supportive care interventions, to people with cancer in order to reduce the demand for clinical resources while maintaining optimal clinical and health outcomes.

Nutrition and physical activity interventions are vital components of cancer care [3]. Prevalence studies demonstrate that as many as 40% of cancer patients are affected by malnutrition, which is associated with increased mortality and health care costs and poor health-related quality of life (HRQoL) [4-6]. Sarcopenia, the loss of skeletal muscle mass, strength, or function, is present in 25%-57% of cancer patients and is an independent predictor of survival [7-9]. Nutrition and exercise interventions can improve muscle mass, muscle strength, physical function, nutritional status, fatigue, and HRQoL for people undergoing cancer treatment [4,5,10,11]. Preliminary evidence also shows potential for improved survival from nutrition interventions delivered throughout treatment [12,13]. Conversely, obesity after treatment completion is associated with reduced cancer survival; nonetheless, less than 20% of cancer survivors meet the dietary recommendations and less than 50% meet the physical activity recommendations, demonstrating a clear role for interventions to support healthy eating behaviors and increased physical activity [14-19]. Nutrition and physical activity interventions, particularly in survivors of breast cancer and men with prostate cancer treated with androgen-deprivation therapy, have been shown to produce clinically meaningful, beneficial weight loss or improvements in muscle mass and cardiometabolic health outcomes (eg, reduced insulin resistance and low-density lipoprotein cholesterol levels) [20,21]. Further, exercise training following a cancer diagnosis has a protective effect on cancer-specific mortality, cancer recurrence, and all-cause mortality [10].

Technology-based platforms such as the internet, mobile phone or tablet apps, and telehealth and wearable devices provide a unique opportunity to deliver broad-reaching interventions and health care to people with cancer. In the general population, technology-supported nutrition and physical activity interventions have demonstrated positive, albeit modest, benefits of increasing physical activity levels, reducing dietary fat intake, and increasing fruit and vegetable consumption [22-24]. In cancer populations, a recent systematic review and meta-analysis investigated digital health behavior-change interventions, which targeted diet and physical activity in cancer survivors [25]. The authors reported a mean improvement of 41 minutes per week of moderate-to-vigorous physical activity levels (*P*=.006) and a pooled reduction in body mass index (BMI)/weight of –0.23 (*P*=.011) with the use of digital interventions. However, technology-supported interventions may still require considerable facilitation by a health professional and use of clinical resources, inhibiting their practicality within usual care [26]. In the context of the growing demand for supportive health care in the cancer setting, technology-supported nutrition and physical activity interventions that are primarily self-guided have the potential to deliver broad-reaching interventions using minimal clinician resources. “Self-guided” refers to interventions delivered with minimal or no facilitation by a health professional and is a key component for improving sustainable dietary and physical activity behavior change. However, the efficacy of self-guided nutrition and physical activity interventions for people with cancer is yet to be established. This systematic review aims to describe and appraise the literature on the efficacy of technology-supported self-guided nutrition and physical activity interventions for people with cancer.

## Methods

### Reporting Guidelines

This systematic review was performed in accordance with the reporting requirements of the PRISMA statement [27]. The protocol was registered in the PROSPERO database with the reference number CRD42017080346 [28].

### Search Strategy

The following databases were searched for peer-reviewed, English-language papers from 1973 to July 2018: Medline Complete, Scopus, CINAHL, EMBASE, Cochrane Library, and SPORTDiscus. The search terms included (Cancer OR oncology OR tumour OR malignancy OR malignant neoplasm) and (online OR internet OR “web-based” OR website OR ehealth OR app OR apps OR application OR mobile application OR smartphone OR mobile phone OR cell phone OR Mhealth OR telehealth OR telemedicine OR technology) and (nutrition OR diet OR physical activity OR exercise). Reference lists of relevant articles were manually searched to identify additional articles.

### Selection Criteria

Studies were eligible for inclusion if they were original research studies on adult participants aged ≥18 years who were diagnosed with any type of cancer at any stage prior to, during, or after cancer treatment, including cancer survivors, and received any treatment modality. Studies were included if they investigated a technology-supported nutrition and physical activity intervention that was largely self-guided and if the technology was accessed primarily outside the clinical setting. An intervention was deemed self-guided when there was minimal or no facilitation by a clinician. Minimal facilitation could encompass activities such as occasional email reminders, an introductory session on navigating the technology platform, or initial exercise prescription. Technology platforms for intervention delivery could be online, mobile phone, or tablet apps or wearable technology. The intervention content needed to focus on nutrition and physical activity. For the purpose of this paper, physical activity also includes exercise interventions. Interventions that included nutrition or physical activity as part of a broader suite of lifestyle or wellness interventions were excluded, unless nutrition or physical activity was a major component of the intervention, comprising at least a quarter of the content together. Studies were required to have a comparator group that could include active controls such as usual care, waitlist controls, or no treatment. Pilot studies that focused on feasibility and acceptability alone were excluded. Outcomes were any measures focused on health-related (eg, quality of life and fatigue), clinical (eg, weight and body composition), health service (eg, resource utilization, hospital admissions, and patient satisfaction), behavioral (eg, dietary intake and physical activity level), or financial outcomes (eg, cost to patients).

### Data Extraction and Quality Assessment

Titles and abstracts were screened for eligibility by two independent reviewers (NK and BB) to exclude articles that were clearly irrelevant. Full-text articles were retrieved and the selection criteria were applied independently by the same two reviewers. Any discrepancies were resolved by discussion.

Each included study was assessed for bias independently by the first author and one of the other authors using the Cochrane Risk of Bias tool [29]. Seven categories were examined to assess selection bias, performance bias, detection bias, attrition bias, reporting bias, and other sources of bias and rated as high risk, low risk, or unclear risk. Ratings were compared and a consensus was reached through discussion. As some of the categories were open to interpretation, all authors agreed on the following: (1) a rating of low risk for performance bias as the nature of these interventions indicated that blinding was not possible and the findings were not likely to be influenced by the lack of blinding, unless another reason was identified to rate otherwise and (2) in the absence of blinding, detection bias was rated as low risk if the outcomes were objectively assessed, and as high risk if outcomes were behavioral or patient reported. All studies were included regardless of the bias rating. The Behaviour Change Taxonomy by Michie et al (2013) was used to describe the type of behavior-change techniques reported within each included study [30]. The heterogeneity of studies and diversity of outcomes indicated that the quantitative synthesis of data was not appropriate.

## Results

### Study Selection

**Figure 1:**
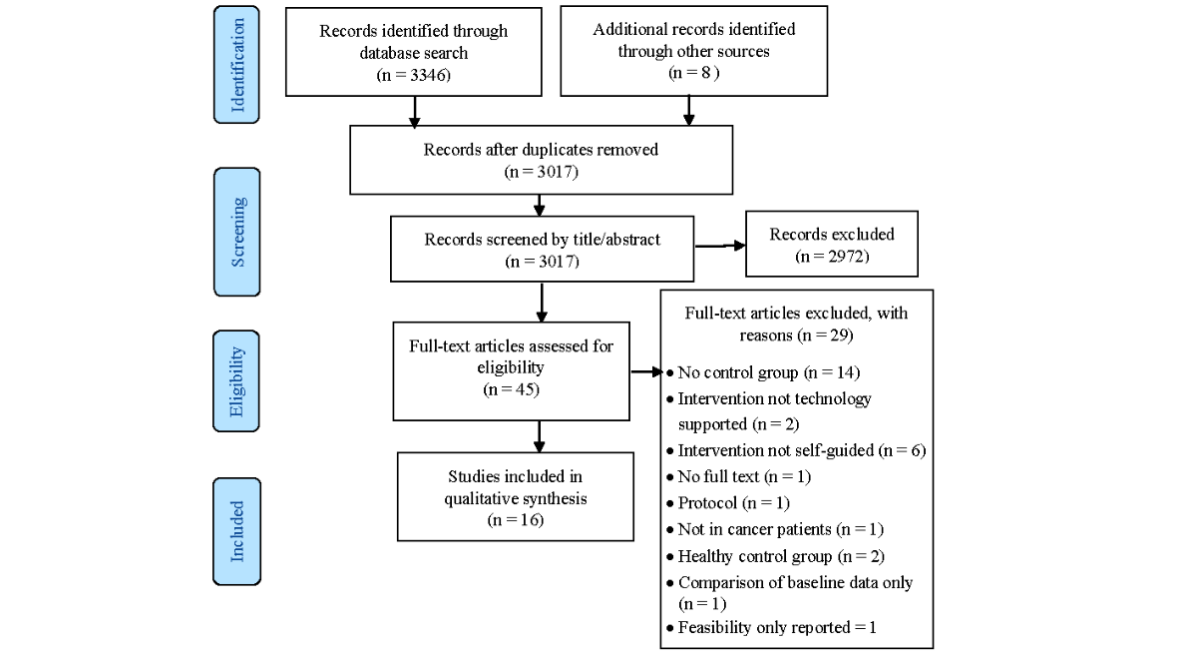
Selection process.

The literature search identified a total of 3346 articles, and eight more articles were identified from the reference lists of relevant papers. Of the total, 18 articles representing 16 studies were included in the systematic review. Two studies were reported in two separate papers each. One of these studies reported on different outcomes in separate papers [31,32]; the other study reported on outcomes at 6 months [33] and 12 months [34] in separate papers. All articles are included, but the findings are presented as a single study. Figure 1 describes the selection process.

### Overview of Included Studies

Tables 1 and 2 present the characteristics of all included studies. All studies were randomized controlled trials presenting data from 2684 participants. The duration of follow-up ranged from 10 weeks to 12 months, and five studies reported follow-up duration of ≥6 months. Comparator groups included waitlist controls in six studies [32,35-38], active controls in five studies [39-43], usual care in four studies [33,44-46], and no treatment controls in one study [47]. Three studies used more than one comparator group, one of which used two active controls [45], one used a no-treatment control and two active controls [46], and one used a waitlist control and an active control [37]. 

**Table 1:** Characteristics of the included studies.

Author, year, country	Participants	Study design	Behavior-change technique^a^	Intervention		Type of control group
				Self-guided component	Facilitated component	
Bantum et al, 2014, United States [36]	Cancer survivors between 4 wk and 5 y posttreatment N=352 Consent rate=56% Retention rate=86%	Two-arm RCT^b^	Goal setting (behavior) Feedback on behavior Self-monitoring of outcome(s) of behavior Social support (unspecified) Instructions on how to perform a behavior Social comparison Credible source	Six web-based modules of 22 topics, including healthy eating, exercise, stress management, communication, and fatigue management, accessed over 6 weeks	Baseline training of the website content and weekly goal setting of health behavior change was discussed with a facilitator on the website	Waitlist control
Forbes et al, 2015, Canada [35]	Breast or prostate cancer survivors N=95 Consent rate=32% Retention rate=88%	Two-arm RCT	Goal setting (behavior) Problem solving Feedback on behavior Self-monitoring of behavior Social support (unspecified) Instructions on how to perform a behavior Social comparison Credible source Social reward	Nine web-based exercise behavior-change modules, sequentially published over a 9-week period, including exercise safety, goal setting, benefits, barriers, and strategies on compliance to exercise. Participants logged step counts on an accelerometer website	Weekly email updates about new information and brief summary of previous week PA^c^ level	Waitlist control
Galiano-Castillo et al, 2016 & 2017, Spain [31,32]	Breast cancer survivors N=81 Consent rate=89% Retention rate=88%	Two-arm RCT	Feedback on behavior Instructions on how to perform a behavior Demonstration of the behavior Credible source	Web-based 8-week intervention providing access to 24 exercise sessions, 3 per week, tailored to participants, including warm-up, resistance and aerobic exercise, and cool down to meet The American College of Sports Medicine recommendations for cancer survivors	Baseline familiarity with the content to individualize the exercise training. Instant messages and video conference were available if requested by the participant for further exercise support	Waitlist control
Gnagarella et al, 2016, Italy [39]	Any cancer diagnosis during or after treatment N=125 Consent rate=28% Retention rate=59%	Two-arm RCT	Instructions on how to perform a behavior Information about health consequences Credible source	Web-based 6-month intervention with access to weekly forums, blogs, and content on healthy eating to reduce treatment symptoms, control weight loss, or maintaining body mass and guidelines for healthy eating	Option to interact with facilitator upon request	Active control
Kanera et al, 2016 & 2017, Netherlands [33,34]	Cancer survivors between 4 wk and 56 wk posttreatment N=462 Consent rate=36% Retention rate=82.5%	Two-arm RCT	Goal setting (behavior) Problem solving Action planning Discrepancy between current behavior and goals Feedback on behaviors Self-monitoring of behaviors Instructions on how to perform a behavior Information about health consequences Social comparison Credible source Pros and cons	Web-based intervention over 6 months with 8 modules of videos, written content, goal setting, action planning, and problem identification including nutrition, exercise, smoking, fatigue, anxiety, and depression	No facilitation	Usual care
Krebs et al, 2017, United States [44]	Breast or prostate cancer survivors N=86 Consent rate=53% Retention rate=79%	Two-arm RCT	Goal setting (behavior) Problem solving Feedback on behavior Social support (unspecified) Instructions on how to perform a behavior Credible source	DVD^d^-based intervention over 12 weeks of nutrition and exercise advice, derived from the American Cancer Society guidelines for cancer survivors. Components included enhancing knowledge, developing positive expectations, reducing barriers, and supporting self-efficacy	No facilitation	Usual care
Lee et al, 2014, South Korea [40]	Breast cancer survivors N=59 Consent rate=50% Retention rate=97%	Two-arm RCT	Goal setting (behavior) Problem solving Action planning Discrepancy between current behavior and goal Feedback on behavior Self-monitoring of behavior Instructions on how to perform the behavior Information about health consequences Credible source	Web-based 12-week intervention with participants encouraged by SMS^e^ to access the tailored Web-based content biweekly, which covered enhancing exercise and diet behaviors, barriers and diet, or exercise guidelines for cancer survivors	30-min training on using the Web-based platform	Active control
Mayer et al, 2014, United States [49]	Colon cancer survivors between 6 wk postoperation and 12 mo postdiagnosis N=284 Consent rate=54% Retention rate=80%	Two-arm RCT	Goal setting (behavior) Self-monitoring of behavior Social support (unspecified) Instructions on how to perform a behavior Credible source	Six-month mobile app physical activity intervention including skill building, information provision, and support services designed to increase daily activity levels. New content added over the period of the intervention	Personal trainer available to answer questions in discussion groups and initiate individually tailored private messages to inactive participants	Control
Ormel et al, 2018, Netherlands [48]	Any cancer diagnosis during active systemic treatment or survivorship N=32 Consent rate=53% Retention rate=96%	Two-arm RCT	Self-monitoring of behavior Prompts/cues Credible source	12-wk mobile app physical activity intervention consisting of physical activity advice and self-monitoring	No facilitation	Usual care
Pope et al, 2018, United States [41]	Breast cancer survivors who completed treatment 3 mo to 10 y prior N=30 Consent rate=77% Retention rate=67%	Two-arm RCT	Problem solving Social support (unspecified) Instructions on how to perform a behavior Demonstration of the behavior Credible source	10-wk smart watch and Facebook intervention including a strength and aerobic training program plus access to a private Facebook page for delivery of health education tips	Tutorial on the use of the smart watch and Facebook page. Participants contacted by researchers every other week to encourage continuation of the intervention	Active control
Rabin et al, 2011, United States [42]	Young adult cancer survivors (18-39 y) N=18 Consent rate=72% Retention rate=94%	Two-arm RCT	Goal setting (behavior) Feedback on behavior Self-monitoring of behavior Social support (unspecified) Instructions on how to perform the behavior Information about health consequences Credible source	Web-based 12-wk intervention with access to purposefully designed website providing information on weekly goal setting and PA resources individually tailored to the stage of change and feedback generated from monthly questionnaires	One-off induction to the website	Active control
Sajid et al, 2016, United States [45]	Men aged ≥65 y with prostate cancer on hormone therapy N=19 Consent rate, not reported Retention rate=68%	Three-arm RCT	Self-monitoring of behavior Instructions on how to perform the behavior Demonstration of the behavior Graded tasks	Intervention arm 1: Wearable device-based 6-wk intervention plus instructions for safe aerobic and tailored resistance training exercise, and total steps per day Intervention arm 2: Wearable device plus an individually tailored Wii-Fit exercise program for 6 wk	Both arms: 45-min exercise introduction with an accredited exercise physiologist and weekly reminders for completion of diary and pedometer assessments	Control
Uhm et al, 2017, South Korea [43]	Breast cancer survivors N=356 Consent rate, not reported Retention rate=95%	Two-arm RCT	Feedback on behavior Self-monitoring Instructions on how to perform the behavior Graded tasks	Mobile app and wearable device-based 12-wk intervention. Content and instructive videos with PA goals were available on the mobile app based on a 2MWT^f^ at baseline. Content on the mobile app was additional to the facilitated exercise program	Physiatrists prescribed the amount of 90-150 min of moderate-intensity aerobic exercise and 4-8 resistance training exercises	Active control
Vallance et al, 2007, Canada [46]	Breast cancer survivors N=377 Consent rate=25% Retention rate=89.7%	Four-arm RCT	Self-monitoring of behavior Instructions on how to perform the behavior Credible source	All groups were instructed to perform 30-min MVPA^g^ 5 d/wk over a 12-wk intervention. Intervention arm 1: Wearable device and documents to record daily step total Intervention arm 2: Wearable device plus self-perusal of guidelines for exercising among breast cancer survivors and a document to record daily step total Intervention arm 3: Self-perusal of guidelines for exercising among breast cancer survivors	No facilitation	Control
Valle et al, 2017, United States [37]	Breast cancer survivors N=35 Consent rate=70% Retention rate=94%	Three-arm RCT	Goal setting (behavior) Problem solving Goal setting (outcome) Feedback on behavior Self-monitoring of behavior Monitoring of outcome(s) of behavior without feedback Instructions on how to perform a behavior Information about health consequences Graded tasks Credible source Self-talk	Intervention arm 1: Web-based with mobile companion app providing 24-wk intervention on nutrition and PA for body weight and weekly email of standardized content on behavior-change strategies related to weight loss. Web or mobile app accessed to log body weight and PA Intervention arm 2: Same as intervention arm 1 plus a wearable device and additional education on steps per day and meeting the steps-per-day recommendations	Both intervention arms: 1-h face-to-face nutrition consult, 24 weekly emails of behavior change to reach 150-225 min of moderate-intensity PA and for energy reduction by 100 kcal through dietary intake, and tailored feedback on weight were provided	Waitlist control
Yun et al, 2012, Korea [38]	Cancer survivors <24 mo posttreatment N=273 Consent rate=28% Retention rate=89%	Two-arm RCT	Monitoring outcome(s) of behavior by others without feedback Social support (unspecified) Instructions on how to perform a behavior Credible source	Web-based 12-wk intervention via seven education modules with personally tailored information on energy conservation, PA, nutrition, sleep hygiene, pain, distress management, and information on fatigue	No facilitation	Waitlist control

^a^Determined from Michie et al [30].

^b^RCT: randomized controlled trial.

^c^PA: physical activity.

^d^DVD: digital video disk.

^e^SMS: short message service.

^f^2MWT: 2-minute walk test.

^g^MVPA: moderate-to-vigorous physical activity.

Sample sizes ranged from 18 to 462 participants. Thirteen studies included cancer survivors of a variety of cancer types, with a majority of breast or prostate cancer survivors [32,33,35-38,40-44,46,48]; one study included participants receiving active cancer treatment [45]; and two studies included participants who were at any cancer stage prior to, during, or after cancer treatment [39,49].

Nine studies examined a physical activity intervention [32,35,41-43,45,46,48,49], one study examined a nutrition intervention [39], and six studies examined a combined physical activity and nutrition intervention [33,36-38,40,44]. Of the studies that examined a combined intervention, three were part of a broader suite of interventions encompassing areas such as fatigue management, stress management, and pain; however, nutrition and physical activity were major components of these interventions [33,36,38].

Participation rates for the studies ranged from 25% to 89% (median=53%), and four studies reported a consent rate of ≥70%. However, once enrolled in the studies, participant retention was generally high, with 12 of the 16 studies retaining >80% of participants over the study duration. Methods for measuring uptake and adherence to the intervention, including the proportion of patients who accessed the intervention content and the average number of logins to the intervention website, varied considerably, making comparison between studies challenging. Four studies did not report on uptake or intervention adherence [39,43,45,49].

Studies used between 3 and 11 (median=5.5) behavior-change techniques, and provision of instructions to perform a behavior was the most common technique used in all but one of the included studies. Other commonly used techniques included Credible Source to deliver information (14 studies), self-monitor behavior (11 studies), and provide feedback on behavior (9 studies). In some studies, insufficient description of the intervention prevented a full analysis of the behavior-change techniques employed [38,39,46,49]. Insufficient description of the intervention also limited the ability to assess the quality and content of the interventions. However, 14 of the included studies used a behavior change technique—Credible Source—meaning the content was provided by a source that was considered reliable.

**Table 2:** Outcome measures and findings of the included studies.

Author, year, country	Outcomes	Intervention uptake	Between-group findings
Bantum et al, 2014, United States [36]	Measured at baseline and 6 mo Primary outcomes: fruit and vegetable intake, PA^a^, depression, fatigue, and insomnia	67% participants accessed all sessions. Mean number of sessions accessed=5.3 (SD 1.28)	Compared to control group, the intervention group showed a significant improvement in insomnia (9.6 to 10.1 vs 9.6 to 9.2; *P*=.03), strenuous exercise (29 min/wk [no change] vs 32 to 51 min/wk; *P*=.01), stretching (26 to 25 min/wk vs 31 to 46 min/wk; *P*=.01)
Forbes et al, 2015, Canada [35]	Measured at baseline and 10 wk Primary outcome: feasibility of the intervention Secondary outcomes: PA and quality of life	67% viewed the modules at least once. Average number of logins over the 9-wk period=10.3	Improved mental health in the waitlist controls compared to the intervention group (*d*=0.37, *P*=.01)
Galiano-Castillo et al, 2016 & 2017, Spain [31,32]	Measured at baseline, 8 wk, and 6 mo Primary outcomes: strength, quality of life, pain, and fatigue	Adherence rate=93.9% of the scheduled sessions	Compared to the control group, at 8 wk, the intervention group showed improved isometric abdominal (*P*<.001, *d*=1.02), back (*P*<.001, *d*=1.31), lower-body (*P*=.001, *d*=–0.81); and hand grip strength (*P*=.006, *d*=0.66); 6MWT^b^ (*P*<.001, *d*=0.92); global quality of life (*P*<.001, *d*=0.89); physical functioning (*P*<.001, *d*=0.9); role functioning (*P*=.001, *d*=0.78); cognitive functioning (*P*=.002, *d=* 0.75); pain severity (*P*=.001, *d*=–0.82); fatigue (*P*<.001, *d*=–0.89); and pain interference (*P*=.05, *d*=–0.47). At 6 months, the intervention effect was maintained for all except role functioning and pain severity
Gnagarella et al, 2016, Italy [39]	Measured at baseline and 6 mo Primary outcomes: nutrition knowledge, food consumption, and quality of life	Not reported	No significant differences between groups for nutrition knowledge or food consumption. Compared to the control group, the intervention group had improved role functioning in the quality of life scale at 6 mo (–6.3 vs 5.1, *P*=.02)
Kanera et al, 2016 & 2017, Netherlands [33,34]	Measured at baseline, 6 mo, and 12 mo Primary outcomes: PA and dietary behavior	Average of 2.23 (SD 1.53) modules followed. PA activity module followed by 25% participants. Diet module followed by 62% participants	Significantly increased vegetable consumption (*P*=.023) and moderate physical activity (*P*=.04) in the intervention group compared to the control group after 6 mo. This effect was sustained at 12 mo for physical activity but not vegetable consumption
Krebs et al, 2017, United States [44]	Measured at baseline and 12 wk Primary outcomes: fruit and vegetable intake and PA	72% viewed the DVD^c^, 50% completed the full DVD	No significant between-group differences
Lee et al, 2014, South Korea [40]	Measured at baseline and 12 wk Primary outcomes: diet composition and PA Secondary outcomes: quality of life, anxiety, depression, fatigue, motivational readiness, and self-efficacy	89% of patients consistently participated in the program throughout the intervention	Compared to control group, after 12 wk, the intervention group showed significantly improved proportion of participants meeting the recommendations of 150 min/wk moderate-intensity exercise (35.7% vs 65.5%, *P*<.0001) and five servings of fruit or vegetables/d (32.1% vs 55.2%, *P*=.001) and improved the diet-quality index (9.6 vs 11.1, *P*=.001), physical functioning (75.9 vs 83.6, *P*=.02), fatigue (15.3 vs 13.5, *P*=.03), and self-efficacy for exercise (*P*=.02) and fruit and vegetable intake (*P*=.02)
Mayer et al, 2014, United States [49]	Measured at baseline and 3, 6, and 9 mo Primary outcome: physical activity Secondary outcomes: distress and quality of life	93.8% participants described as users (accessed system at least once). Of the 180 days of possible use, mean use=55.3 days (SD 50.0)	No significant differences between the intervention and control groups
Ormel et al, 2018, Netherlands [48]	Measured at baseline, and 6 and 12 wk Primary outcome: feasibility of the intervention Secondary outcome: PA	Not reported	Compared to the control group, the intervention group had significantly increased total minutes of PA at 6 wk (2348 min/wk vs 3773 min/wk, *P*=.04), but there was no difference in sedentary time between groups. There were no significant between-group differences at 12 weeks
Pope et al, 2018, United States [41]	Measured at baseline and 10 wk Primary outcomes: PA and energy expenditure Secondary outcomes: anthropometry, body composition, cardiorespiratory fitness, quality of life, and psychosocial constructs	Participants wore the smart watch 6-7 d/wk and accessed the Facebook page 1.2 times/wk on an average	Compared to the intervention, the control group demonstrated improved physical activity-related social support and reduced barriers. No other significant between-group differences were observed
Rabin et al, 2011, United States [42]	Measured at baseline and 12 wk Primary outcomes: feasibility and acceptability Secondary outcomes: PA, mood, and fatigue	Average number of website logins=14.75 (SD 8.46)	Compared to the control group, at 12 weeks, there was a medium effect of the intervention on increasing MVPA^d^ (16.5 min/wk vs 102.5 min/wk, *d*=0.64) and a large effect of the intervention on mood (–5.00 vs –25.86, *d>* 0.80) and fatigue (–3.30 vs –11.43, *d*>0.80). However, these differences were not statistically significant
Sajid et al, 2016, United States [45]	Measured at baseline, 6 wk, and 12 wk Primary outcome: physical performance Secondary outcomes: steps per day, lean muscle mass, and chess press repetitions	Not reported	Compared to controls, intervention arm 1 showed greater improvement in physical performance (*P*=.04) and a higher rate of change in steps per day at 12 wk (*P*<.01). There were no additional between-group differences seen either between intervention arm 1 and control or intervention arm 2 and control
Uhm et al, 2017, South Korea [43]	Measured at baseline, 6 wk, and 12 wk Primary outcomes: PA, quality of life, anthropometrical measures, body mass index, blood pressure, functional capacity, and hand grip strength	Not reported	There were no significant between-group differences at 6 and 12 wk
Vallance et al, 2007, Canada [46]	Measured at baseline and 12 wk Primary outcomes: PA Secondary outcomes: quality of life, fatigue, brisk walking, and objective step count	Participants who received the written material reported on the content 2.1 times on an average Participants who received the pedometer recorded their steps on 83.3% of study days	Compared to the control group, a significant improvement was seen in MVPA at 12 wk in intervention arm 1 (30 min/wk vs 59 min/wk, *P*=.02) and intervention arm 2 (30 min/wk vs 87 min/wk, *P*=.02). Compared to the control group, significant improvements were seen in brisk walking at 12 wk in intervention arm 1 (0 min/wk vs 94 min/wk, *P<*.001), intervention arm 2 (0 min/wk vs 58 min/wk, *P*=.03), and intervention arm 3 (0 min/wk vs 72 min/wk, *P*=.006) were observed. No differences in objective step count. Intervention arm 2 showed significant improvement in quality of life (6.9 vs 1.1, *P*=.003) at 12 wk compared to control
Valle et al, 2017, United States [37]	Measured at baseline, 3 mo, and 6 mo Primary outcome: feasibility Secondary outcomes: PA, body mass index, weight, body composition, and metabolic syndrome biomarkers	Intervention arm 1: 100% participants reported reading some/all/most of the email content and email feedback Intervention arm 2: 90% participants reported reading some/all/most of the email content and email feedback	Intervention arm 2 significantly reduced body mass index (–0.4 vs 0.1, *P*=0.046) compared to controls at 6 mo. Intervention arm 1 maintained HBA_1c_^e^ levels as compared to increased HBA_1c_ levels observed in the control group (0.0 vs 0.15, *P*=.02). No other significant between-group differences were observed
Yun et al, 2012, Korea [38]	Measured at baseline and 12 wk Primary outcome: fatigue Secondary outcomes: nutritional status, quality of life, anxiety, and depression	Intervention completed by 83.1% participants	Compared to the control group, the intervention group had a significantly greater decrease in fatigue (group difference=–0.66, *P*=.001, *d*=0.29) and anxiety (–0.9, *P*=.004, *d*=0.33) and significantly greater increase in nutritional status (0.47, *P*=.04, *d*=0.23), global quality of life (5.22, *P*=.02, *d*=0.26), emotional functioning (4.69, *P*=.02, *d*=0.19), social functioning (4.73, *P*=.03, *d*=0.24), and cognitive functioning (6.09, *P*=.002, *d*=0.24) after 12 wk

^a^PA: physical activity.

^b^6MWT: six minute walk test.

^c^DVD: digital video disk.

^d^MVPA: moderate to vigorous physical activity.

^e^HBA_1c_: hemoglobin A_1c_.

### Technology Platform and Level of Facilitation

The majority of studies (n=9) were delivered as Web-based modules with content covering nutrition and physical activity recommendations [32,33,35-40,42]. Three studies used a wearable device to deliver a physical activity intervention [41,45,46]. The remaining studies used mobile apps (n=3) or digital video disk (n=1) to deliver content on nutrition and physical activity recommendations [43,44,48,49]. In five studies, there was no facilitation of the self-guided intervention [33,38,44,46,49]. In the remaining studies, facilitation involved baseline training on the intervention or Web platform [32,36,37,40-43,45], emails sent at varying frequencies to provide content updates, reminders or summaries of completed physical activity [35,37,45], and interaction with a facilitator in a discussion group or upon request [39,48].

### Risk of Bias Assessment

The outcome of the bias assessment is presented in Table 3. All included studies were randomized controlled trials. However, as evident from Table 3, there was a high degree of variation in the bias of the included studies, in particular, lack of clarity about the randomization process and few studies included blinding of the outcome assessment.

### Behavioral Outcomes

Of the five studies that investigated dietary behaviors, two found improvements in the intervention arm. Kanera et al (2016) reported a significant increase in vegetable consumption after 6 months of use of a personalized web-based intervention, although this increase was not sustained at 12 months postintervention [33,34]. Similarly, Lee et al (2014) observed a significant improvement in diet quality and fruit and vegetable intake following a 12-week Web-based intervention, but did not measure longer-term outcomes [40].

One of the studies where no benefit of intervention was observed on dietary behavior did not specify the inclusion criteria for selecting participants who were not meeting the dietary recommendations [36]. The authors subsequently found they had recruited participants who had better-than-average fruit and vegetable intake and little need for health behavior change [36]. The two remaining studies that found no improvement in dietary behavior were not powered to detect between-group differences [39,44].

In total, 14 studies examined interventions to promote physical activity behaviors, of which eight studies reported positive outcomes. Bantum et al (2014) reported significant increases in both strenuous exercise and stretching at 6 months after a 6-week Web-based intervention including a suite of health behavior-change content [36]. Another 8-week Web-based intervention delivering physical activity recommendations tailored to participants found significant improvements in isometric abdominal, back, and lower-body muscle strength at 2 and 6 months following the intervention [32]. A 12-week mobile app intervention providing physical activity advice reported a significant improvement in the total minutes of physical activity at 6 weeks but no difference at 12 weeks [49].

Kanera et al (2016) found a significant increase in moderate physical activity levels after 6 months of use of a personalized Web-based intervention; however, similar to the effect on dietary behavior in this study, the difference was not sustained at 12 months [33,34]. In a 12-week Web-based intervention providing tailored exercise content to participants, significant improvements were observed in the proportion of participants following moderate-intensity exercise recommendations [40]. Sajid et al (2016) found a higher rate of change in the number of steps per day among participants who received instructions for exercising and used a wearable device in combination as compared to the control group. However, the same effect was not observed when participants followed a tailored Wii-Fit exercise program in combination with the wearable device, although the sample size was small and not powered to detect differences [45]. In another study, participants using a wearable device alone or in combination with access to exercise guidelines experienced significant improvements in weekly moderate-to-vigorous intensity physical activity and brisk walking [46]. However, improvements in brisk walking were also observed in participants with access to exercise guidelines alone [46]. Although underpowered to detect a significant difference in moderate-to-vigorous physical activity levels, another study reported a medium effect (102.5 [intervention] vs 16.5 [control] minutes/week) of a Web-based intervention after 12 weeks [42].

**Table 3:** Risk of bias for included studies.

Author, year	Random sequence generation	Allocation concealment	Blinding of participants/personnel	Blinding of outcome assessment	Incomplete outcome data	Selective reporting	Other bias
Bantum et al, 2014 [36]	Low	Unclear	Low	High	High	High	High
Forbes et al, 2015 [35]	Low	Unclear	Low	Low	Low	Unclear	Low
Galiano-Castillo et al, 2016 [31], 2017 [32]	Low	Unclear	Low	Low	Low	High	Low
Gnagnarella et al, 2016 [39]	Low	Unclear	Low	High	High	High	High
Kanera et al, 2016 [33], 2017 [34]	Low	Low	High	Low	Low	Low	Low
Krebs et al, 2017 [44]	Low	Low	Low	High	Low	Unclear	Low
Lee et al, 2014 [40]	Low	Low	Low	High	Low	Low	Low
Mayer et al, 2018 [49]	Unclear	Unclear	Low	High	Low	Unclear	Low
Ormel et al, 2018 [48]	Low	Unclear	Low	High	Low	Low	Low
Pope et al, 2018 [41]	Low	Unclear	High	Low	High	Unclear	Unclear
Rabin et al, 2011 [42]	Unclear	Low	Low	High	High	Unclear	Unclear
Sajid et al, 2016 [45]	Unclear	Low	Low	Unclear	Unclear	Unclear	High
Uhm et al, 2017 [43]	High	Low	Low	High	High	Unclear	Low
Vallance et al, 2007 [46]	Low	Low	Low	High	Low	High	Low
Valle et al, 2017 [37]	Low	Low	Low	Low	Low	Low	Unclear
Yun et al, 2012 [38]	Low	Low	Low	High	Low	Low	Unclear

Several studies that did not report positive outcomes had limitations. Although Forbes et al (2015) did not specifically select participants who did not meet the physical activity recommendations, a sub-group analysis showed that their Web-based intervention was more effective in participants who were not meeting the recommendations [35]. Similarly, another study failed to select participants with poor physical activity behaviors [41], and two studies were not powered to detect an effect [37,44]. Uhm et al used an active control group that received the same information as the intervention group but without the support of a mobile app [43]. Mayer et al (2018) considered the possibility that the lack of benefit was due to the short duration of the intervention [48]; however, the 6-month intervention was longer than that included in several studies reporting positive outcomes [32,36,40,49].

Overall, technology-supported self-guided interventions appeared to have some benefit on dietary intake; however, the few studies that assessed this outcome had several limitations. A relatively consistent positive benefit was noted for physical activity, although the long-term benefits remain unknown; only one study reported outcomes beyond 6 months and found no effect at that time point. There were no patterns in the type or number of behavior-change techniques supporting the effective interventions in comparison to those for which no effect was observed. Effectiveness of interventions for physical activity level did not depend on whether physical activity was patient reported or objectively measured.

### Clinical Outcomes

Clinical outcomes were reported in four of the included studies [37,38,41,43]. One study examined weight change, lean body mass, fat mass, BMI, and metabolic syndrome biomarkers after 6 months of use of a Web- and mobile app-based nutrition and physical activity intervention [37]. The intervention was tested in two groups: one used the intervention alone and other used the intervention along with wearable technology. The results showed an improvement in the BMI in the latter intervention group as compared to the control group. However, only the group that used the intervention alone showed an improvement in metabolic syndrome biomarkers [37]. Another study using a mobile app intervention and wearable device showed no difference in BMI, arm circumference, handgrip strength, and blood pressure between the intervention group and an active control group that received written information [43]. A further study reported a significant improvement in the nutritional status, measured using the Mini Nutrition Assessment tool, at 12 weeks after the use of a combined Web-based nutrition and physical activity intervention [38]. Pope et al (2018) found no differences in weight or body composition using a 10-week smart watch and Facebook physical activity intervention [41].

Overall, changes in clinical outcomes as a result of technology-supported self-guided interventions were inconsistent but were assessed in only a small number of studies, making it difficult to draw any concrete conclusions.

### Health-Related Outcomes

HRQoL was assessed in nine studies, five of which used the European Organisation for Research and Treatment of Cancer Quality of Life Questionnaire - Core 30 [32,38-40,43], three used the Functional Assessment of Cancer Therapies questionnaire [35,38,48], and one used the Patient-Reported Outcome Measurement System [41]. Two studies reported improved global HRQoL [32,38], and four studies reported improvements in one or more of the HRQoL subscales [32,38-40]. Studies demonstrating the most-beneficial HRQoL outcomes tended to be Web-based, used waitlist controls, involved a physical activity intervention, and reported very high adherence to the intervention. A further three-arm randomized trial by Sajid et al (2016) investigating functional capacity alone through use of a short physical performance battery reported improved physical performance in the wearable device intervention group compared to the control group, but this result was not observed for the wearable device plus Wii Fit arm [45].

Fatigue was measured in six studies, three of which used the Brief fatigue Inventory [36,38,40] and the remaining studies used the Piper Fatigue Scale [32], the fatigue scale from the Functional Assessment of Cancer Therapy-Anemia measurement system [46], and the Profile of Mood States scale [42]. Of the six studies, four reported significantly improved fatigue following the intervention [32,38,40,42]. Of the four studies, one study involved a 12-week Web-based intervention with modules covering sleep, hygiene, pain, distress management, and fatigue in addition to nutrition and physical activity. As such, the positive effect on fatigue may have been related to other components of the intervention. Two of the six studies found no significant difference in fatigue [36,46]. No consistent differences were evident between the studies that did and did not observe an effect on fatigue.

Yun et al (2012) observed a significantly greater decrease in anxiety, but not depression, after their 12-week Web-based intervention. However, similar to the effect on fatigue, the effect on anxiety may have been related to other components of the intervention beyond nutrition and physical activity [38]. Four studies on physical activity or combined nutrition and physical activity interventions measured depression [36,40], anxiety [40], distress [48], or mood disturbance [42], and found no significant between-group differences.

One study assessed the effect of a Web-based intervention of modules covering nutrition, exercise, stress management, communication, and fatigue management on insomnia and found a significant improvement following the 6-week intervention [36]. One study reported significant improvement in pain severity after an 8-week Web-based physical activity intervention as compared to the control arm [32].

Overall, HRQoL and fatigue appeared to improve after technology-supported self-guided interventions, and the majority of studies showed a positive benefit. The effects on mental health, pain, and insomnia require further investigation.

No studies investigated health service use or financial outcomes. In addition, no studies examined or compared outcomes according to key potential moderating factors such as gender, socioeconomic position, or ethnicity. Studies including only female patients with breast cancer were as likely to report positive outcomes as those including both male and female participants.

## Discussion

The main finding of this review was that technology-supported self-guided interventions appear to improve physical activity behaviors and fatigue in people with cancer in the short term. Although many studies only measured these outcomes in the short term, a few studies that assessed these outcomes in the longer term found that this benefit was not sustained. There was a minor effect of such interventions on dietary behavior and an inconsistent effect on clinical outcomes such as weight, BMI, body composition parameters, and mental health outcomes. This finding is largely consistent with that of previous systematic reviews investigating digital health or eHealth interventions in cancer survivors, without focusing on the self-guided component [25]. For instance, Roberts et al (2017) reported improved physical activity and BMI in cancer survivors through digital health physical activity and diet interventions [25]. Similarly, Haberlin et al (2018) observed improvement in physical activity following the use of eHealth to promote physical activity among cancer survivors [50]. Both these reviews concluded that the effect of these interventions was promising, but studies using objective measures and assessing the impact on long-term outcomes remain a priority. Similar conclusions were drawn in a systematic review of computer-tailored physical activity and dietary behavior-promotion programs in the general population; this review showed improvements in physical activity and dietary behavior, but these improvements were limited to the short or medium term due to the lack of long-term follow-up in studies [23].

Inconsistent reporting of self-guided interventions is an issue that has resulted in a recent recommendation for the development of a standardized reporting framework for these types of interventions in people with cancer [51]. Overall, self-guided interventions are not well described in the literature, which limits definitive extraction of the key components of the intervention associated with improved health outcomes in people with cancer. In particular, this affected the extraction of the behavior-change techniques used within the interventions, and in some studies, sufficient information was not reported to determine the specific strategies used. A further issue was the reporting of intervention compliance, which was measured in different ways across studies and at times, not reported at all. Therefore, in cases where interventions did not report positive outcomes, it was difficult to determine whether the intervention itself was not effective or whether the lack of effect was related to poor compliance or insufficient participant engagement in the intervention. Similar issues were identified in a 2017 systematic review on self-guided interventions for psychosocial distress in people with cancer [26]. Of note, in 2011, a CONSORT checklist was published to improve the reporting of Web-based and mobile health interventions [52]; however, none of the studies in this systematic review reported the use of this framework.

Two of the studies included in this review did not specifically select participants who had poor health behaviors for the study. However, in their dietary and physical activity intervention group, Bantum et al (2014) found that they had recruited participants who had a better-than-average fruit and vegetable intake, and thus, there was no need to change their dietary behavior, potentially explaining the lack of effect of the intervention [36]. Similarly, Forbes et al (2015) did not target recruitment to participants who were not meeting physical activity recommendations; however, a subsequent subanalysis revealed that the intervention was more effective in participants who did not meet the physical activity recommendations at baseline [35]. Although only two studies in our review discussed this limitation, the eligibility criteria of the included studies revealed that 10 of the 16 studies did not specifically target participants with poor dietary or physical activity behaviors. Failure to specifically recruit participants requiring supportive care interventions has also been identified as an issue in several previous systematic reviews of supportive care interventions in cancer [26,53]. It is imperative that future research in this area targets people who require support but are currently not able to access the care they require.

Technology-supported self-guided interventions require a high level of self-motivation for participants to engage with the intervention. Compliance, or intervention uptake, provides some indication of participant engagement. However, inconsistencies in how the engagement was reported, as previously discussed, lead to difficulties in comparing studies. Although measured post-intervention, and therefore, not a true indicator of engagement with the intervention, patient satisfaction provides some insight into the acceptability of an intervention, which has implications for adherence and engagement [54]. A number of the studies included in this review measured patient satisfaction using a questionnaire or interview. Although patient satisfaction was high, there was no consistent approach or questionnaire used across studies; as such, comparison between studies was not possible.

Of the 16 studies included in this review, only two involved patients who were undergoing active cancer treatment. Personalized nutrition and physical activity interventions delivered individually by a clinician or other health professionals during cancer treatment have demonstrated benefits on body composition, nutritional status, quality of life, fatigue, and functional outcomes [4,5,20,55]. However, considering the increasing incidence of cancer, a personalized approach to nutrition and physical activity interventions may have limited long-term feasibility. Models for stratifying patients by risk categories are proposed for the management of cancer survivors [56] and may need to be considered for interventions delivered during active treatment. The level of facilitation in the studies included in this review was minimal, making this a potential cost-effective approach, although evaluation of the cost effectiveness was not a feature of any of the studies and resource requirements were not reported.

The strengths of this review are reporting according to the PRISMA guidelines; inclusion of risk of bias assessment; and categorization of the behavior change techniques underpinning the interventions, which has not been included in previous systematic reviews. Limitations include substantial heterogeneity among the included studies in terms of sample size, risk of bias, outcome measures, type and duration of interventions, and use of behavior-change techniques, which restricted our ability to distinguish the components of the interventions that were effective and to complete our meta-analyses.

In summary, this systematic review identified a short-term benefit of technology-supported self-guided interventions with regard to physical activity behavior and fatigue and a small benefit with regard to dietary behavior and HRQoL in people with cancer. However, there was considerable heterogeneity in the quality of the included studies and some heterogeneity along with major methodological limitations, which make interpretation of the findings challenging. Despite the potential of technology-supported interventions, there is a lack of evidence for their long-term benefit, which requires further investigation. Furthermore, a high proportion of studies did not actively target people with poor nutrition or physical activity behaviors. Future studies should ensure that interventions are tested in people requiring improvements in nutrition and physical activity who are not currently able to access the care they require.

## Abbreviations

2MWT: two minute walk test
6MWT: six minute walk test
BMI: body mass index
DVD: digital video disk
HBA_1c_: hemoglobin A_1c_
HRQoL: health-related quality of life
MVPA: moderate to vigorous physical activity
PA: physical activity
RCT: randomized controlled trial
SMS: short message service
